# Prenatal arsenic exposure alters the placental expression of multiple epigenetic regulators in a sex-dependent manner

**DOI:** 10.1186/s12940-019-0455-9

**Published:** 2019-02-28

**Authors:** Emily F. Winterbottom, Yuka Moroishi, Yuliya Halchenko, David A. Armstrong, Paul J. Beach, Quang P. Nguyen, Anthony J. Capobianco, Nagi G. Ayad, Carmen J. Marsit, Zhigang Li, Margaret R. Karagas, David J. Robbins

**Affiliations:** 1 Molecular Oncology Program, DeWitt Daughtry Family Department of Surgery, University of Miami Miller School of Medicine, Miami, FL 33136 USA; 2 Department of Epidemiology, Geisel School of Medicine at Dartmouth, Hanover, NH 03755 USA; 3 Pulmonary and Critical Care Medicine, Dartmouth-Hitchcock Medical Center, Lebanon, NH 03756 USA; 4 Center for Therapeutic Innovation, Department of Psychiatry and Behavioral Sciences, The Miami Project to Cure Paralysis, Sylvester Comprehensive Cancer Center, University of Miami, Miller School of Medicine, Miami, FL 33136 USA; 5 Department of Environmental Health, Rollins School of Public Health at Emory University, Atlanta, GA 30322 USA

**Keywords:** Arsenic, Prenatal, Epigenetic, Histones, PRDM6, Sex, Fetal placenta

## Abstract

**Background:**

Prenatal exposure to arsenic has been linked to a range of adverse health conditions in later life. Such fetal origins of disease are frequently the result of environmental effects on the epigenome, leading to long-term alterations in gene expression. Several studies have demonstrated effects of prenatal arsenic exposure on DNA methylation; however the impact of arsenic on the generation and decoding of post-translational histone modifications (PTHMs) is less well characterized, and has not been studied in the context of prenatal human exposures.

**Methods:**

In the current study, we examined the effect of exposure to low-to-moderate levels of arsenic in a US birth cohort, on the expression of 138 genes encoding key epigenetic regulators in the fetal portion of the placenta. Our candidate genes included readers, writers and erasers of PTHMs, and chromatin remodelers.

**Results:**

Arsenic exposure was associated with the expression of 27 of the 138 epigenetic genes analyzed. When the cohort was stratified by fetal sex, arsenic exposure was associated with the expression of 40 genes in male fetal placenta, and only 3 non-overlapping genes in female fetal placenta. In particular, we identified an inverse relationship between arsenic exposure and expression of the gene encoding the histone methyltransferase, PRDM6 (*p* < 0.001). Mutation of *PRDM6* has been linked to the congenital heart defect, patent ductus arteriosus.

**Conclusions:**

Our findings suggest that prenatal arsenic exposure may have sex-specific effects on the fetal epigenome, which could plausibly contribute to its subsequent health impacts.

**Electronic supplementary material:**

The online version of this article (10.1186/s12940-019-0455-9) contains supplementary material, which is available to authorized users.

## Background

Arsenic is a natural contaminant of air, water, and soil. High levels of groundwater arsenic contamination, exceeding 100–150 μg/L, have been consistently associated with a range of disease conditions, including respiratory and pulmonary disease, skin lesions, and various cancers [1], and effects have also been observed with lower levels of exposure (e.g., [2, 3]). Prenatal arsenic exposure has been linked to spontaneous abortion, infant mortality and preterm birth, low birth weight, and increased rates of infant infections, as well as chronic health problems in later life [1]. High levels of arsenic exposure tend to be limited to isolated regions of countries such as Mexico, Bangladesh, and Taiwan. However, millions of people worldwide, including residents of some US states, are exposed to levels of arsenic that are lower, but still frequently exceed the World Health Organization’s recommended limit of 10 μg/L [4], and the health effects of such exposure are not yet clear. The New Hampshire Birth Cohort Study (NHBCS) was initiated in 2009 with the goal of determining how environmental contaminants, including arsenic, affect the health of pregnant women and their infants [5]. Women exposed to low to moderate levels of arsenic through the use of private wells, as well as through dietary sources, were enrolled in the study. Analyses of data from the NHBCS have revealed associations between prenatal arsenic exposure and measures of fetal and newborn growth [5, 6]; rates of infant infections [7]; immune profiles, inflammatory markers, and leptin levels in cord blood [8–10]; and gene expression and DNA methylation in fetal placenta [11–15]. Thus, it is increasingly evident that even low-to-moderate levels of arsenic have multiple effects on the developing fetus and lead to adverse infant health outcomes. However, the mechanisms that link arsenic’s molecular effects with its impacts on infant health are still being determined.

Differential gene expression, in various tissues, developmental stages, or disease states, is controlled by epigenetic mechanisms. Hence, epigenetics plays an essential role in normal cellular function and homeostasis. Moreover, environmental effects on the epigenome occurring prenatally are thought to persist through to adulthood, causing altered susceptibility to various diseases, a phenomenon known as “developmental origins of health and disease” (DOHaD). The epigenome includes methylation of the cytosine bases of DNA; post-translational histone modifications (PTHMs); and miRNA-mediated regulation of gene expression. Exposure to arsenic has been shown in several studies, both in vivo and in vitro, to affect each of these components (reviewed in [16]). Importantly, this suggests that prenatal exposure to arsenic can have long-term effects on future health, which is in line with observed associations between such exposure and increased susceptibility to a range of chronic health conditions, including cardiovascular and respiratory disease, and cancer [1].

The best-characterized forms of PTHM include methylation, acetylation, phosphorylation and ubiquitination of various residues within different histones. These modifications influence gene expression positively or negatively by generating binding sites for chromatin-modifying enzymes or affecting nucleosome structure/positioning. Arsenic exposure has been shown to lead to gene-specific and global changes to multiple PTHMs, in both in vitro and epidemiological studies (reviewed in [16]). The aim of the current study was to determine the effects of prenatal exposure to arsenic on the fetal expression of genes that encode key regulators of PTHMs, using samples from the fetal portion of the placenta. Most of these PTHM regulators can be classed as “writers” (e.g., histone methyltransferases and acetyltransferases), “erasers” (e.g., histone demethylases/deacetylases), “readers” of these histone modifications (e.g., bromodomain-containing proteins), or chromatin “remodelers”. Understanding the way in which arsenic affects these regulators will provide clues as to some of the epigenetic changes that may occur in fetal tissues in response to arsenic exposure, and the mechanisms underlying these changes. Further, our findings may provide insights into the mechanisms by which prenatal exposure contributes to subsequent disease.

## Methods

### Study cohort

This study analyzed data from 311 pregnant mothers and their infants, who were recruited to the New Hampshire Birth Cohort Study (NHBCS) [5] between February 2012 and September 2013. The NHBCS is a longitudinal study designed to evaluate the effects of prenatal exposure to toxicants such as arsenic. Women eligible for enrollment sought prenatal care at one of the study clinics in New Hampshire and obtained their household water from private wells, which are a potential source of arsenic exposure in this region. Participants were aged 18–45, English-speaking, mentally competent, and pregnant with a singleton infant. The mothers’ demographic and lifestyle information, and household water samples, were collected during prenatal visits, and pregnancy outcome and infant clinical characteristics were taken from medical records. The study was approved by The Committee for the Protection of Human Subjects at Dartmouth College.

### Urine sample collection and arsenic measurement

Details of urine sample collection and analysis have been described previously [5]. Briefly, spot urine samples were taken at a second trimester prenatal visit (approximately 24–28 weeks of gestation), collected into acid-washed containers containing diammonium diethyldithiocarbamate, and frozen at − 80 °C until analysis. Samples were analyzed for individual arsenic species (arsenite [AsIII], arsenate [AsV], monomethylarsonic acid [MMAV] and dimethylarsinic acid [DMAV], and arsenobetaine) by high-performance liquid chromatography inductively coupled plasma mass spectrometry at the University of Arizona. Detection limits ranged from 0.11 to 0.13 μg/L for the individual arsenic species. Numbers of samples below the detection limits for each urinary arsenic species were as follows: AsIII: 168 (54.02%); AsV: 186 (59.81%); MMAV: 90 (28.94%); DMAV: 3 (0.96%)**.** In such instances, the level of that species was recorded as the detection limit divided by the square root of 2. Total maternal urinary arsenic (U-As) was calculated as the sum of the individual arsenic species excluding arsenobetaine, which is considered to be non-toxic.

### Placenta biopsy and gene profiling

At the time of delivery, biopsies measuring 1 cm deep and 1–2 cm across were taken from the fetal portion of the placenta, at the base of the umbilical cord insertion. Care was taken to avoid maternal decidua, fibrous connective tissue and calcifications. Samples were immersed in RNAlater (Life Technologies) and stored at − 80 °C until analysis. RNA was extracted using an RNA/DNA extraction kit (Norgen Biotek, Thorold, ON) and quantified using a NanoDrop spectrophotometer. Of a total of 349 placental samples obtained, fifteen samples gave low RNA yields. RNA quality analysis was performed on the remaining 334 samples using an Agilent BioAnalyzer 2100, and we selected the 312 samples with the highest RIN (RNA integrity number) scores (≥3.67) for gene expression analysis. One sample was subsequently found to lack urinary arsenic data and was excluded from final analyses. Gene expression analysis was performed using the NanoString system (NanoString Technologies) at the Oncogenomics Core Facility at the University of Miami. The Nanostring probes were designed to detect a subset of 138 epigenetic proteins in the “writer”, “reader”, “eraser”, “remodeler”, or “other” classes (Additional file 1), which were chosen for analysis based on their prior utilization by the Ayad laboratory [17], as well as their reported roles in development and disease. Probes to detect 5 housekeeping genes (*ACTB*, *GAPDH*, *HPRT1*, *RPL0* and *RPL19*) were also used. Internal positive control probes, which are designed by Nanostring to target sequences defined by the External RNA Controls Consortium (ERCC), were included in each assay at varying concentrations. Raw count data was normalized to the geometric mean of the internal positive controls and that of the housekeeping genes, using nSolver software. Negative control probes, which target absent sequences defined by the ERCC, were also included in each assay. Among the probes tested, *KDM4DL, PRDM9, PRDM12, PRDM13, PRDM14, SMYD1, UTY* and *WIF1* had average counts below the combined average count of the negative control probes, indicating that these genes have low expression in fetal placenta. Among these, only *PRDM13* appeared to be significantly associated with arsenic exposure in female placenta and the full cohort in our analysis.

### Statistical analysis

Using the normalized count data, multivariable linear regression models were used to identify associations between candidate gene expression and maternal U-As levels, modeling natural log-transformed gene expression as a function of natural log-transformed U-As. Models were adjusted for maternal age at enrollment, as this was found to be a potentially confounding factor in our previous analyses of the NHBCS [14, 15]. We also tested the effect of adjusting our results for urinary creatinine, as a measure of urine dilution. Urinary creatinine data were available for 265 (85.2%) of the 311 participants. For the vast majority of genes, this adjustment did not change the directionality of the coefficients for log transformed U-As, and all standardized coefficients were within 0.1 standard error of the corresponding coefficient obtained in the unadjusted model when the same subset of participants was analyzed (Additional file 2). Further, a goodness of fit test (F-test) indicated that adding creatinine to the models did not significantly improve goodness of fit (Additional file 2). For these reasons, and to optimize our statistical power, we opted to not include adjustment for urinary creatinine in our final statistical models. All analyses were conducted using the R statistical program (http://cran.r-project.org/).

## Results

The study cohort consisted of 311 mother-infant dyads, who were enrolled in the NHBCS. Demographic information is provided in Table 1. The average age of the mother at delivery was 31.8 years, and the average gestational age at birth was 39.4 weeks. Only a small percentage of the mothers (5.8%) reported smoking during pregnancy. Forty-seven percent of the mothers were overweight or obese (BMI ≥ 25) before pregnancy. About half the infants were male, and the average birth weight was 3.4 kg. The median total arsenic level in the mothers’ urine at mid-gestation was 3.7 μg/L (IQR 2.2–6.3 μg/L).

**Table 1:** Study cohort demographic information

Characteristic	Mean (SD)
Gestational age (wks)	39.4 (1.5)
Maternal age at enrollment (yrs)	31.8 (4.8)
Parity	0.93 (1.1)
Pre-pregnancy BMI (kg/m^2^)	26.3 (5.9)
Ever smoked during pregnancy	18 (5.8)
Infant birth weight (kg)	3.4 (0.5)
Infant sex:	
Male	163 (52.4)^a^
Female	148 (47.6)^a^
Total urinary arsenic (U-As, μg/L)	3.7 (2.2–6.3)^b^

^a^number (%); ^b^median (interquartile range)

**Fig. 1:**
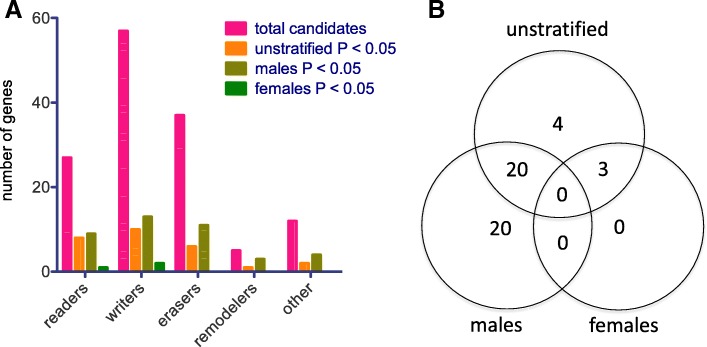
Epigenetic candidate genes significantly associated with arsenic exposure. Multivariable linear regression analysis of associations between epigenetic candidate gene expression and U-As levels. **a**) Categorization of candidate epigenetic genes. Graph shows the total number of candidate genes in each category, and the number in each category associated with U-As levels in the unstratified cohort, male placentas, or female placentas. **b**) Venn diagram showing the number of candidate epigenetic genes whose expression significantly associated with U-As levels, at a significance level of *P* < 0.05, in the unstratified cohort, male placentas, and/or female placentas. Analyses were adjusted for maternal age at enrollment

Multivariable linear regression analysis showed that, among our epigenetic regulator candidate genes, the placental expression of 27 genes was statistically significantly associated with U-As levels at the *P* < 0.05 level (Additional files 2A and 3). These included members of each class of epigenetic regulator (Fig. 1a). Only one of these associations, with *PRDM6* (*PR/SET Domain 6*), was inversely related (Additional files 2A and 3, Table 3). No associations remained statistically significant after FDR adjustment for multiple comparisons (Additional file 2A).

Next, since we have previously identified considerable sex-dependence in the relationship between placental gene expression and arsenic exposure [13, 14], we analyzed the associations of our candidate genes with U-As levels after stratifying the cohort by infant sex. We found that the candidate genes associated with arsenic exposure in male and female fetal placentas were completely non-overlapping (Fig. 1b and Additional files 2B-C, 4, 5), in keeping with our previous findings. Moreover, while a total of 40 candidate genes were associated with arsenic exposure in male fetal placentas, only 3 genes showed associations in females. All of the statistically significant associations in males were positive, while in females, two significant associations were positive and one, with *PRDM6*, was negative. In males, the statistically significant genes included members of each class of epigenetic regulators, although a slightly disproportionate number of these encoded remodelers (Fig. 1a). Again, no statistically significant associations were retained after FDR adjustment for multiple comparisons (Additional file 2B-C).

Many epigenetic regulators act as part of multiprotein complexes in which the non-catalytic subunits serve to localize, activate, or stabilize the catalytic subunits. To determine how such complexes might be affected by arsenic exposure, we used the EpiFactors database [18] to identify complexes containing the candidate genes that were associated with U-As. In female fetal placenta, none of the three genes that were significantly associated with U-As were known components of epigenetic complexes. However, in males, 15 of the 40 U-As-associated genes were components of one or more epigenetic complex. Among these, four complexes—the COMPASS-like MLL3, 4 complex [19], the NuA4-related complex [20, 21], and two SWI/SNF complexes: BRM and BRM_BRG [19]—were each represented by three U-As-associated genes (Table 2). These included the core catalytic subunit of SWI/SNF complexes, SMARCA4 (BRG1), and those of MLL3/4 complexes: KMT2D (MLL4), KMT2C (MLL3), and KDM6A (UTX). These data suggest that such complexes may be particularly active in arsenic-exposed placenta, altering the epigenetic landscape. However, it should be noted that some components from each complex were missing from our candidate gene list, and so it is unclear whether all the subunits of any particular complex were influenced by arsenic exposure.

**Table 2:** Epigenetic complexes represented by upregulated genes in arsenic-exposed male fetal placenta

Complex	Components upregulated in male placenta	Function	References
SWI/SNF_BRM-BRG	PBRM1, BRD7, SMARCA4	ATP-dependent chromatin remodeling	[19]
COMPASS-like MLL3, 4	KMT2D, KMT2C, KDM6A	H3K4 methylation and H3K27 demethylation	[19]
SWI/SNF_BRM	SMARCA4, HDAC2, HDAC1	ATP-dependent chromatin remodeling (by homology)	UniProt, [19]
NuA4-related	SRCAP, EP400, BRD8	Histone H4 and H2A acetylation (by homology)	UniProt, [20, 21]

The EpiFactors database [18] was used to identify epigenetic regulatory complexes, the expression of whose components in male fetal placenta was positively associated with U-As. Complexes containing three U-As-associated genes are shown

Interestingly, among the candidate genes, only *PRDM6* (PR/SET domain 6), showed a significant negative association with U-As levels in the unstratified cohort, and in females alone (Table 3, Additional files 2, 3, 4). Moreover, this association had the strongest negative beta coefficient estimate in all analyses, including in male placenta (Table 3, Additional files 2, 3, 4, 5).

**Table 3:** Association of U-As with *PRDM6* expression in fetal placenta

	Beta coefficient estimate	*P* value	FDR-adjusted *P* value
Unstratified	−0.138	< 0.001	0.066
Males	−0.116	0.066	0.185
Females	−0.156	0.002	0.246

## Discussion

In this study, we examined the effect of prenatal exposure to arsenic, at common environmental levels, on the fetal expression of key epigenetic regulators. We looked for associations between the expression of 138 candidate epigenetic genes in the fetal placenta and maternal U-As levels, as a measure of fetal arsenic exposure. We found that the expression of these genes was associated with arsenic exposure in a highly sex-dependent manner: 40 candidates were associated with U-As in male placenta, while only three non-overlapping genes had associations in females. These results are consistent with our previous work, in which we found that the expression of a candidate set of developmental and stemness genes in the fetal placenta was associated with U-As levels in a sex-specific manner [14]. Indeed, it is well established that the placentas associated with male and female fetuses show distinct molecular responses to a range of environmental stresses (reviewed in [22]). Further, several epidemiological and animal studies have demonstrated that arsenic has a range of sex-specific effects on the epigenome of various tissues [23–30], including differential DNA methylation of umbilical cord blood and fetal placenta following prenatal arsenic exposure [11, 12, 28, 29]. We note that a higher percentage of fetuses in our cohort were male; thus, it is possible that lower statistical power might have affected the ability to detect statistically significant associations in females versus males. However, we note that the observed associations were entirely non-overlapping, which suggests that biological differences rather than variability were responsible for our findings.

Among these previous studies, two focused on PTHMs in peripheral blood mononuclear cells from a study of adults in Bangladesh exposed to arsenic through their drinking water [23, 24]. Sex-specific associations between arsenic exposure and various PTHMs were identified. Of particular interest, histone 3 lysine 4 trimethylation (H3K4me3) and histone 3 lysine 27 trimethylation (H3K27me3) were positively associated with arsenic levels in drinking water in females, but negatively associated in males [24]. In our study, we found positive associations between arsenic exposure and the expression of three components of the COMPASS-like MLL3, 4 complex in male placenta. This complex is thought to catalyze both trimethylation of H3K4, and demethylation of H3K27 [19]. Thus, our results are partially in keeping with the previous report, which showed a negative association between arsenic exposure and H3K27 methylation in males. However, the association with H3K4 methylation is the opposite of what would be expected based on our data. In contrast, a study of male steel workers exposed to arsenic in air identified a positive association between exposure levels and H3K4me2, which is consistent with our results [31]. Such differences may be related to the level of arsenic exposure or the type of tissue analyzed.

Among our candidate genes, we observed the strongest negative association with *PRDM6* and higher arsenic exposure, a finding that was statistically significant in females. This suggests that arsenic may suppress the fetal expression of the *PRDM6* gene, and particularly when the fetus is female. *PRDM6* encodes a member of the PR/SET domain family of proteins, which encode histone lysine methyltransferases. It is reported to promote the differentiation of endothelial cells [32], but inhibit that of smooth muscle cells, instead maintaining their proliferation [33]. A recent study found that various independent loss-of-function mutations in PRDM6 were associated with patent ductus arteriosus (PDA) [34]. PDA is a congenital heart defect in which the ductus arteriosus, which channels blood from the pulmonary artery to the aorta, bypassing the lungs, fails to close at birth. PDA is associated with impaired lung development and hypertension, and if untreated, can lead to congestive heart failure and increased infant mortality [35]. In vitro analyses indicated that PRDM6, which is expressed in the smooth muscle of the ductus arteriosus, is important for maintaining this tissue in a proliferative state, and that its loss leads to premature differentiation of these cells and thus interferes with proper ductus closure [34]. The association of increased arsenic exposure with reduced expression of *PRDM6* in our results suggests that prenatal exposure to arsenic may increase the risk of PDA via inhibition of *PRDM6*. Consistent with this hypothesis, increased incidence of PDA (also called ductus Botalli persistens) was associated with maternal arsenic exposure through drinking water in a study from Hungary [36], and with increased particulate matter air pollution (of which arsenic is a common constituent) in Atlanta, USA, and Lanzhou, China [37, 38]. Interestingly, PDA is consistently reported to be 2–3 times more common in female infants than in males (e.g., [39]). Our data show a stronger association of arsenic exposure with *PRDM6* expression in females, suggesting a possible mechanism for this outcome.

It is also interesting to consider the potential effects of reduced *PRDM6* expression in the fetal placenta itself. Based on its reported expression patterns and functions in other tissues, PRDM6 might influence the development and function of the placental villus vasculature. Alterations in this tissue have been associated with multiple adverse pregnancy outcomes including intrauterine growth restriction and impaired infant neurodevelopment [40], suggesting that the observed reduction in placental *PRDM6* expression may have a range of adverse effects on infant health.

Our aim in this study was to use gene expression analysis of the fetal placenta, a fetal tissue that is uniquely accessible for research, to provide clues as to the epigenetic changes that may be occurring not only in the fetal placenta, but also in other fetal tissues. Since arsenic passes readily across the placenta [41], such tissues will be subject to similar levels of exposure. However, we acknowledge that not all the effects we observe will be generalizable to other fetal tissues. Thus, our hypotheses should be followed up by further studies. We chose to use the NanoString system for expression analysis of a set of predefined candidate genes encoding epigenetic regulators. We chose this approach because of the greater power of such technology to detect subtle changes in gene expression, compared to a transcriptome-wide screen. However, our study is consequently not exhaustive, and it is possible that additional epigenetic regulatory genes, that were excluded from our candidate list, may also be affected by arsenic exposure. This was a relatively small study; thus, it should be noted that none of the associations we observed remained significant after adjustment for multiple comparisons. Moreover, we were not able to examine whether the observed changes in gene expression at increased arsenic exposure influenced health outcomes. In particular, since the usual incidence of PDA is only 1 in 2000 births, and exposure to arsenic levels > 10 μg/L was previously reported to increase this incidence by only 75% [36], our study cohort is too small to detect an association of PDA with arsenic exposure or *PRDM6* gene expression. However, our results suggest a possible mechanism by which arsenic exposure contributes to PDA, which may be confirmed by future studies.

## Conclusions

Our findings suggest that arsenic exposure is related to multiple, sex-dependent changes to the epigenome of the fetal placenta, which may also be relevant to other fetal tissues. The consequences of such changes to the long-term health of the child, and how they may mediate arsenic’s known health effects, will be important to pursue in future work.

## Additional files

Additional file 1: Candidate genes used in this study (XLSX 60 kb)

Additional file 2: Associations of candidate gene expression in fetal placenta with maternal U-As. Multivariable linear regression analysis was performed to determine the association between log-transformed maternal U-As and log-transformed fetal placental expression of epigenetic candidate genes, in A) the unstratified cohort, B) males, and C) females. Analyses were adjusted for maternal age at enrollment. *P* values before and after FDR adjustment are shown (columns C-D). The effect of adjustment for urinary creatinine was also determined, by comparing the standardized beta values obtained from the subset of participants for which urinary creatinine data were available (“standardized_subset_beta”, column P) with the equivalent values after adjustment for urinary creatinine (“standardized_subset_beta”, column Q). Results of goodness-of-fit tests are also shown (columns S-T). (XLSX 124 kb)

Additional file 3: Associations of fetal placental candidate gene expression with maternal U-As. Graphical representation of associations between maternal U-As and fetal placental expression of epigenetic candidate genes in the unstratified cohort. Analysis was adjusted for maternal age at enrollment. Dots depict coefficient estimates and error bars represent 95% CIs. Significant associations are those with 95% CIs not crossing zero (red dotted line). (PDF 133 kb)

Additional file 4: Associations of candidate gene expression in female fetal placenta with maternal U-As. Graphical representation of associations between maternal U-As and expression of epigenetic candidate genes in female fetal placentas. Analysis was adjusted for maternal age at enrollment. Dots depict coefficient estimates and error bars represent 95% CIs. Significant associations are those with 95% CIs not crossing zero (red dotted line). (PDF 133 kb)

Additional file 5: Associations of candidate gene expression in male fetal placenta with maternal U-As. Graphical representation of associations between maternal U-As and expression of epigenetic candidate genes in male fetal placentas. Analysis was adjusted for maternal age at enrollment. Dots depict coefficient estimates and error bars represent 95% CIs. Significant associations are those with 95% CIs not crossing zero (red dotted line). (PDF 102 kb)

## Abbreviations

IQR: Interquartile range
NHBCS: New Hampshire Birth Cohort Study
PTHM: Post-translational histone modification
U-As: Total maternal urinary arsenic concentration (excluding arsenobetaine)
