# Supplementary material for: Prenatal arsenic exposure alters the placental expression of multiple epigenetic regulators in a sex-dependent manner
Source: Environ Health. 2019 Feb 28;18:18. doi: 10.1186/s12940-019-0455-9 (PMC6396530; doi:10.1186/s12940-019-0455-9)

Additional file 4. Associations of candidate gene expression in female fetal placenta with maternal U-As.

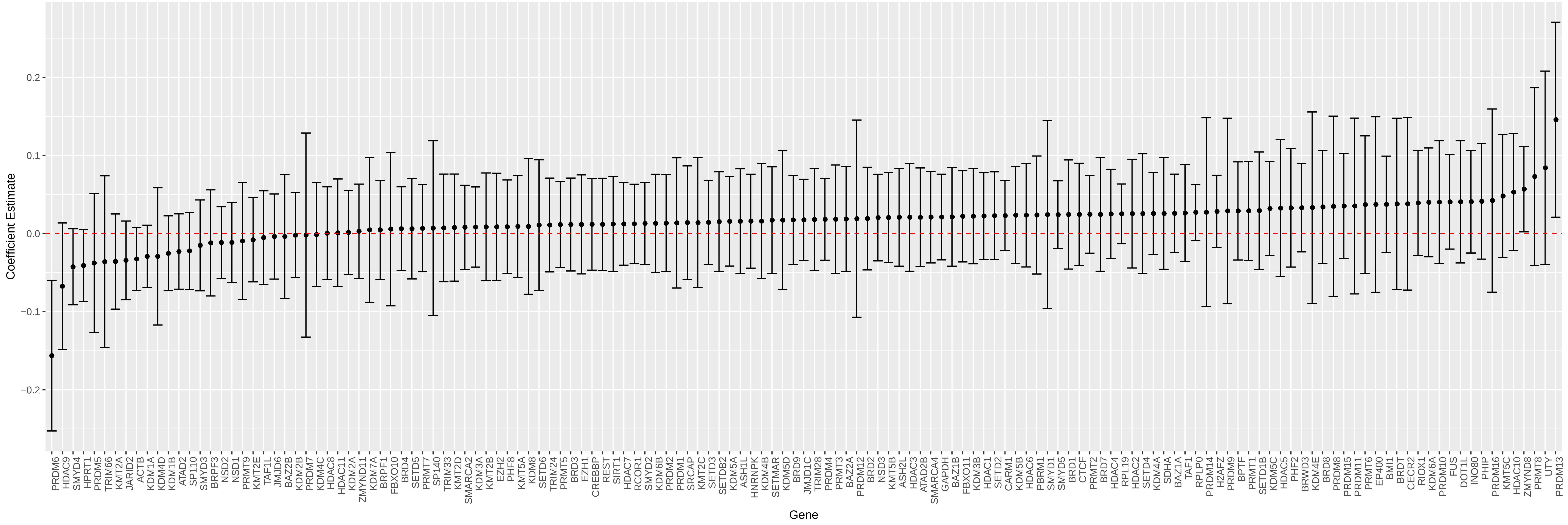

Supplement: Supplementary file 4 — Associations of candidate gene expression in female fetal placenta with maternal U-As. Graphical representation of associations between maternal U-As and expression of epigenetic candidate genes in female fetal placentas. Analysis was adjusted for maternal age at enrollment. Dots depict coefficient estimates and error bars represent 95% CIs. Significant associations are those with 95% CIs not crossing zero (red dotted line). (PDF 133 kb) [file 12940_2019_455_MOESM4_ESM.pdf]
